# Supplementary material for: Transcription elongation can be sufficient, but is not necessary, to advance replication timing
Source: EMBO Rep. 2026 Mar 24;27(8):1964–99. doi: 10.1038/s44319-026-00735-2 (PMC13121604; doi:10.1038/s44319-026-00735-2)
Supplement: Supplementary file 2 — Source data Fig. 1 [file 44319_2026_735_MOESM2_ESM.zip › Fig1/1A/README_1A.rtf]

The sequence files of the insertion casettes. These reporter cassettes were inserted using CRISPR with gRNAs Ptn_5_Insertion_Guide and Ptn_3_Insertion_Guide.
