# Supplementary material for: Transcription elongation can be sufficient, but is not necessary, to advance replication timing
Source: EMBO Rep. 2026 Mar 24;27(8):1964–99. doi: 10.1038/s44319-026-00735-2 (PMC13121604; doi:10.1038/s44319-026-00735-2)
Supplement: Supplementary file 2 — Source data Fig. 1 [file 44319_2026_735_MOESM2_ESM.zip › Fig1/1C/README_1C.rtf]

Bedgraph files to plot replication timing profiles available at GEO GSE310795.For all repli-seq data in this paper, script to process fastq file to bedgraph is available at https://github.com/jlt3/ERCEs_2023 (Ethan, do you have your own?)P-values to show the statistically significant difference in RT between WT casteneus and modified 129 alleles provided here. 
