# Supplementary material for: Transcription elongation can be sufficient, but is not necessary, to advance replication timing
Source: EMBO Rep. 2026 Mar 24;27(8):1964–99. doi: 10.1038/s44319-026-00735-2 (PMC13121604; doi:10.1038/s44319-026-00735-2)
Supplement: Supplementary file 3 — Source data Fig. 2 [file 44319_2026_735_MOESM3_ESM.zip › Fig2/2A/README_2A.rtf]

This Piggy Back transposable element insertion is described in Brueckner et al. EMBO J. 2020 Mar 16;39(6):e103159. doi: 10.15252/embj.2019103159. 
