# Supplementary material for: Transcription elongation can be sufficient, but is not necessary, to advance replication timing
Source: EMBO Rep. 2026 Mar 24;27(8):1964–99. doi: 10.1038/s44319-026-00735-2 (PMC13121604; doi:10.1038/s44319-026-00735-2)
Supplement: Supplementary file 3 — Source data Fig. 2 [file 44319_2026_735_MOESM3_ESM.zip › Fig2/2BCD/README_2BCD.rtf]

The processed (bedgraph) files and raw data (fastq) files to reproduce these plots  are available at GEO GSE310795.The lists of constitive/developmentally regulated early/late replicating regions are available at https://doi.org/10.6084/m9.figshare.24168963.
