# Supplementary material for: Transcription elongation can be sufficient, but is not necessary, to advance replication timing
Source: EMBO Rep. 2026 Mar 24;27(8):1964–99. doi: 10.1038/s44319-026-00735-2 (PMC13121604; doi:10.1038/s44319-026-00735-2)
Supplement: Supplementary file 4 — Source data Fig. 3 [file 44319_2026_735_MOESM4_ESM.zip › Fig3/3C/README_3C.rtf]

Bedgraph files to plot Bru-seq signals available at GEO GSE310676.
