# Supplementary material for: Transcription elongation can be sufficient, but is not necessary, to advance replication timing
Source: EMBO Rep. 2026 Mar 24;27(8):1964–99. doi: 10.1038/s44319-026-00735-2 (PMC13121604; doi:10.1038/s44319-026-00735-2)
Supplement: Supplementary file 6 — Source data Fig. 5 [file 44319_2026_735_MOESM6_ESM.zip › Fig5/5D/README_5D.rtf]

Bedgraph files to plot Bru-seq signals available at GEO GSE310676.Note: Data for 24hr time point is identical for Fig. 4B dox 200 micro gram/mL condition, thus omitted. 
