# Supplementary material for: Transcription elongation can be sufficient, but is not necessary, to advance replication timing
Source: EMBO Rep. 2026 Mar 24;27(8):1964–99. doi: 10.1038/s44319-026-00735-2 (PMC13121604; doi:10.1038/s44319-026-00735-2)
Supplement: Supplementary file 7 — Source data Fig. 6 [file 44319_2026_735_MOESM7_ESM.zip › Fig6/6D/README_6D.rtf]

ATAC-seq data have been previously published and are available from GEO GSE241376.
