# Supplementary material for: Transcription elongation can be sufficient, but is not necessary, to advance replication timing
Source: EMBO Rep. 2026 Mar 24;27(8):1964–99. doi: 10.1038/s44319-026-00735-2 (PMC13121604; doi:10.1038/s44319-026-00735-2)
Supplement: Supplementary file 8 — Source data Fig. 7 [file 44319_2026_735_MOESM8_ESM.zip › Fig7/7A-G/README_7A-G.rtf]

The original replication timing and Bru-seq  data are identical as used in Fig. 6 and available at GEO GSE310795 and GEO GSE310676.See “Materials and Methods” for the processing procedures.
