# Supplementary material for: Transcription elongation can be sufficient, but is not necessary, to advance replication timing
Source: EMBO Rep. 2026 Mar 24;27(8):1964–99. doi: 10.1038/s44319-026-00735-2 (PMC13121604; doi:10.1038/s44319-026-00735-2)
Supplement: Supplementary file 9 — Expanded View Figures [file 44319_2026_735_MOESM9_ESM.pdf]

Expanded View Figures

Figure EV1. Promoter-specific effects of reporter gene insertion on RT.

(**A**) Genome Browser tracks of genome-parsed Bru-Seq biological replicates for the cell lines with the Promoter-HTK insertions (vector sequences not included), indicating that when transcription is observed to read through the vector gene (most prominently with the CAG promoter, but barely above noise with the mPGK promoter), those reads originate exclusively from the 129 allele containing the ectopic gene insertion. (**B**) Genome Browser tracks of Bru-Seq biological replicates for the cell lines with the Promoter-HTK insertions, showing only the inserted allele for each biological replicate (this data cannot be parsed because the insert resides only on the 129 allele). Top tracks display the read-through expression of the Ptn gene, while the zoomed-in, bottom tracks display the expression of the HTK gene. (**C**) Bar graph comparing HTK and Ptn read-through expression in the Promoter-HTK cell lines. The error bars represent standard error. (**D**) Bar graph of the levels of read through Ptn expression in relation to changes in the RT of the Ptn domain. The error bars for the Ptn expression bar graphs represent standard error and the error bars for the RepliSeq data show the range of the difference among the 50 kb bins between the two replicate experiments.

 © The Author(s)

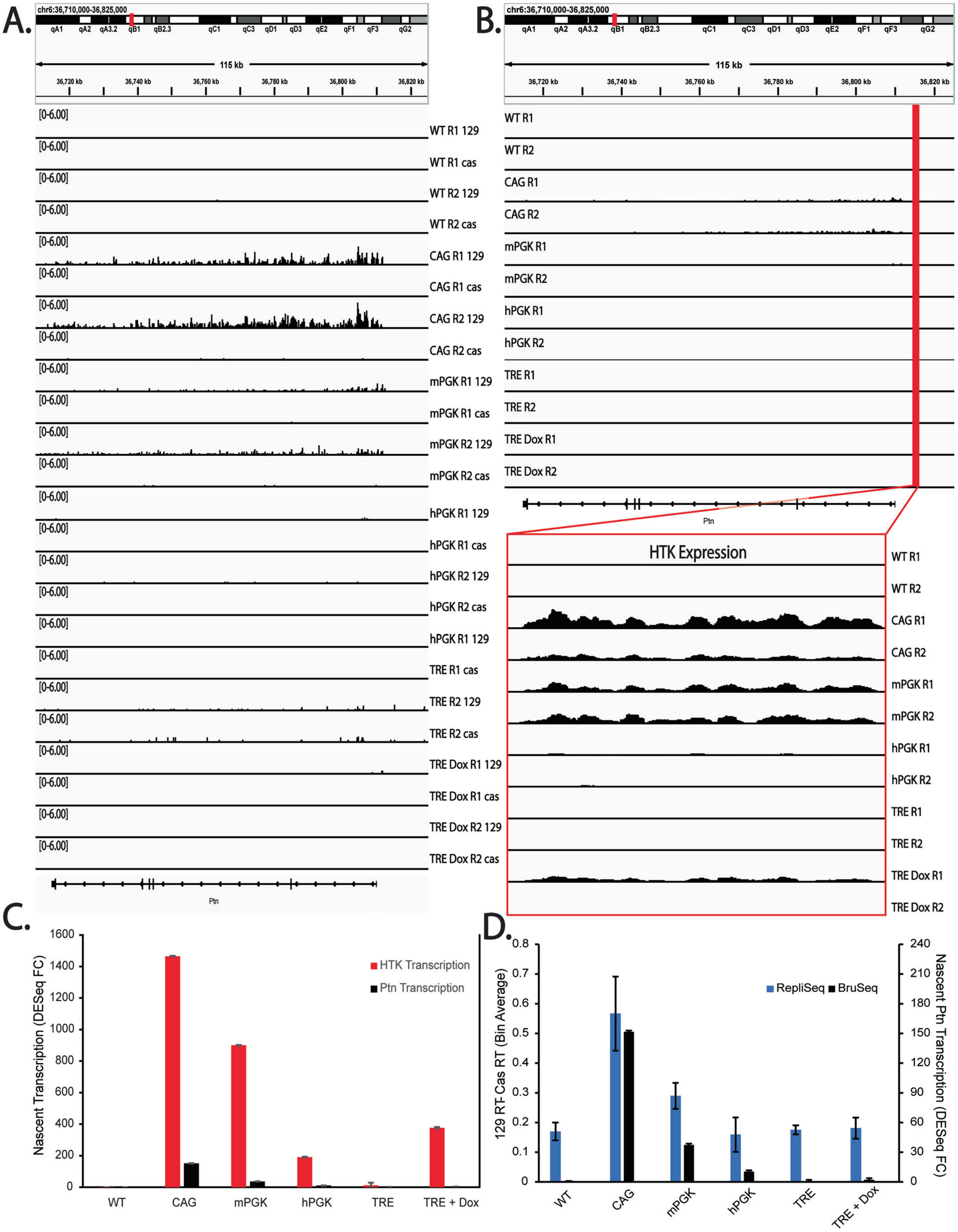
A.
chr6:36,710,000-36,825,000
115 kb
[0-6.00]
WT R1 129
WT R1 cas
WT R2 129
WT R2 cas
CAG R1 129
CAG R1 cas
CAG R2 129
CAG R2 cas
mPGK R1 129
mPGK R1 cas
mPGK R2 129
mPGK R2 cas
hPGK R1 129
hPGK R1 cas
hPGK R2 129
hPGK R2 cas
hPGK R1 129
TRE R1 cas
TRE R2 129
TRE R2 cas
TRE Dox R1 129
TRE Dox R1 cas
TRE Dox R2 129
TRE Dox R2 cas
Ptn
B.
chr6:36,710,000-36,825,000
115 kb
WT R1
WT R2
CAG R1
CAG R2
mPGK R1
mPGK R2
hPGK R1
hPGK R2
TRE R1
TRE R2
TRE Dox R1
TRE Dox R2
Ptn
HTK Expression
C.
Nascent Transcription (DESeq FC)
HTK Transcription
Ptn Transcription
WT
CAG
mPGK
hPGK
TRE
TRE + Dox
D.
129 RT-Cas RT (Bin Average)
Nascent Ptn Transcription (DESeq FC)
RepliSeq
BruSeq
WT
CAG
mPGK
hPGK
TRE
TRE + Dox

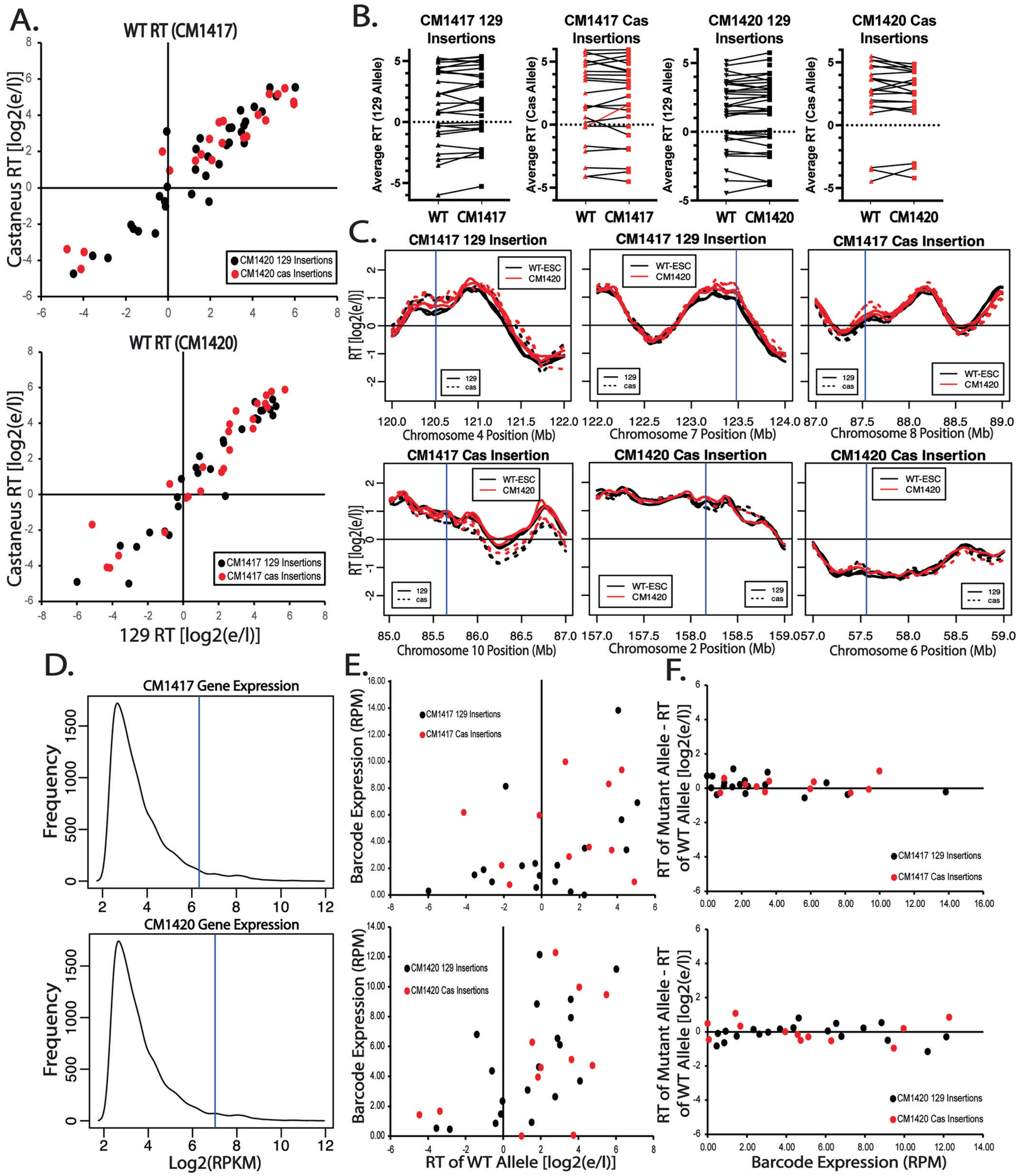
A.
WT RT (CM1417)
CM1420 129 Insertions
CM1420 cas Insertions
WT RT (CM1420)
CM1417 129 Insertions
CM1417 cas Insertions
Castaneus RT [log2(e/l)]
129 RT [log2(e/l)]
B.
CM1417 129 Insertions
CM1417 Cas Insertions
CM1420 129 Insertions
CM1420 Cas Insertions
Average RT (129 Allele)
Average RT (Cas Allele)
WT CM1417
WT CM1420
C.
CM1417 129 Insertion
CM1417 Cas Insertion
CM1420 Cas Insertion
WT-ESC
CM1420
129
cas
RT [log2(e/l)]
Chromosome 4 Position (Mb)
Chromosome 7 Position (Mb)
Chromosome 8 Position (Mb)
Chromosome 10 Position (Mb)
Chromosome 2 Position (Mb)
Chromosome 6 Position (Mb)
D.
CM1417 Gene Expression
CM1420 Gene Expression
Frequency
Log2(RPKM)
E.
Barcode Expression (RPM)
CM1417 129 Insertions
CM1417 Cas Insertions
CM1420 129 Insertions
CM1420 Cas Insertions
RT of WT Allele [log2(e/l)]
F.
RT of Mutant Allele - RT of WT Allele [log2(e/l)]
Barcode Expression (RPM)

 © The Author(s)

Figure EV2. Detailed analysis of PiggyBac vector insertions.

(**A**) Scatter plots of the RT (129 vs Cas) of the WT parental alleles of all CM1417 (left) and CM1420 (right) ectopic insertion sites. Black and red dots represent loci where the eventual insertion was made in the 129 and Cas allele, respectively. (**B**) RT of the CM1417 129 insertions (left), CM1417 Cas insertions (middle-left), CM1420 129 Insertions (middle-right) and CM1420 Cas Insertions (right) before (WT, triangles) and after (CM1417 or CM1420, squares) the PB insertion. There were no statistically significant RT advances, determined as described in Methods, with the exception of the site inserted at Chromosome 8, 87.5 Mb, of the Cas allele of CM1417. The locus with the statistically significant RT change is marked with a red line in the CM1417 plot. (**C**) RT plots of six insertion sites where the largest advances in RT of the insertion allele were observed, including the single statistically significant RT change at the Cas Chromosome 8 allele (top right). (**D**) Distribution of all expressed genes in CM1417 (top) and CM1420 (bottom) clones. The x-axis represents the log2(RPKM) expression level of each gene and the y-axis represents the number of genes that are expressed at each level. RPKM was calculated from the average read count for each gene across two biological replicates. Genes with extremely low or no expression (RPKM < 5) were excluded from the plot. Average GFP expression, calculated by dividing the GFP RPKM of each clone by the number of insertions in that clone, is marked with vertical blue lines. (**E**) Position effect of each insertion locus on the expression level of each insertion. To differentiate between expression levels at each insert, only reads containing the 16-nucleotide barcode were extracted from the unprocessed FastQ and were used to calculate the RPM for each barcode. (**F**) Effect of transcription of individual insertions on the RT of the insertion site. The Y-axis range represents the dynamic range of RT in each clone.

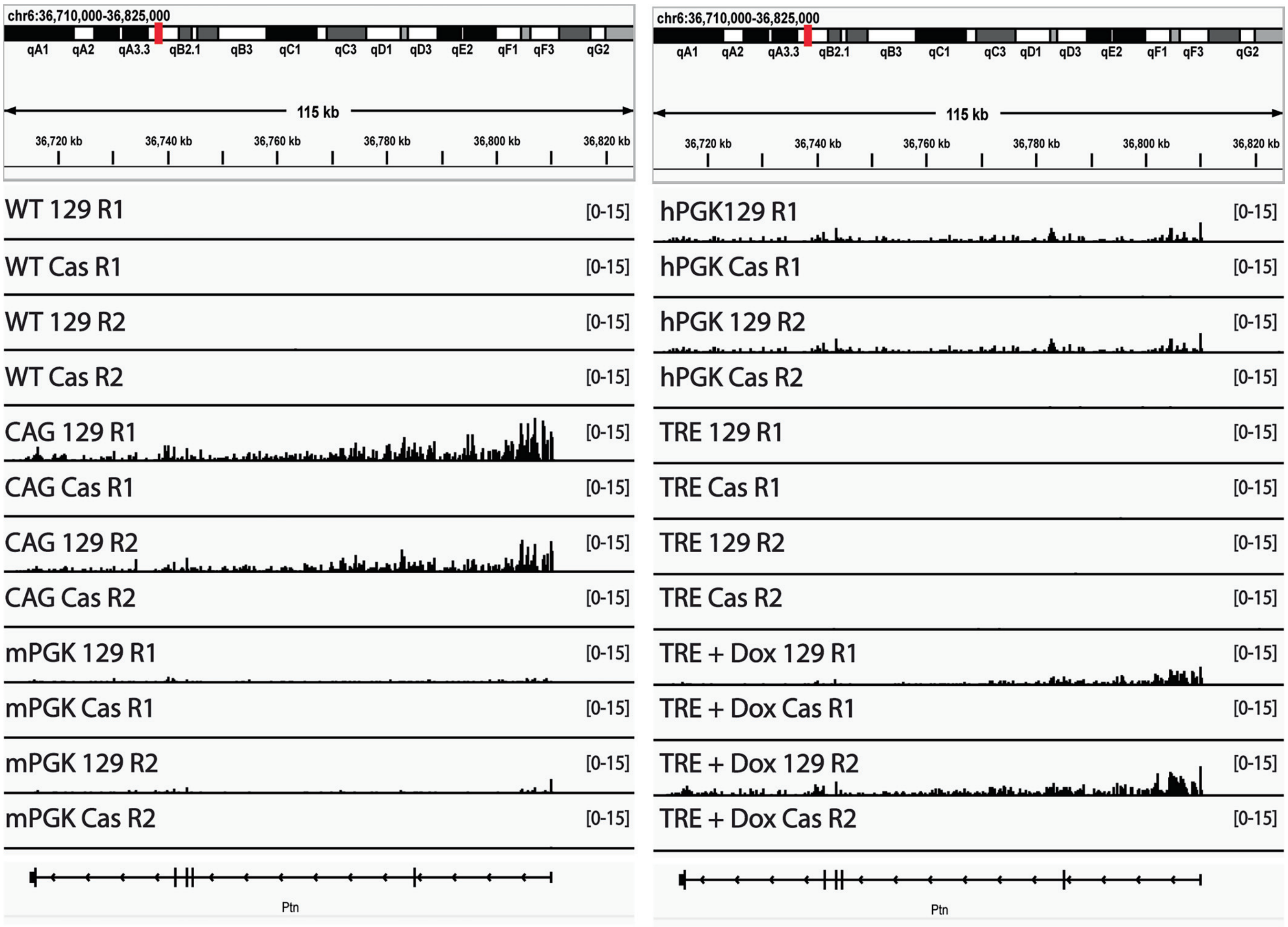

Figure EV3. Transcription of a long transcript enhances the ability of promoters to advance RT.

Genome Browser track of parsed Bru-Seq biological replicates for the cell lines in which the inserted promoters drive Ptn transcription.

 © The Author(s)

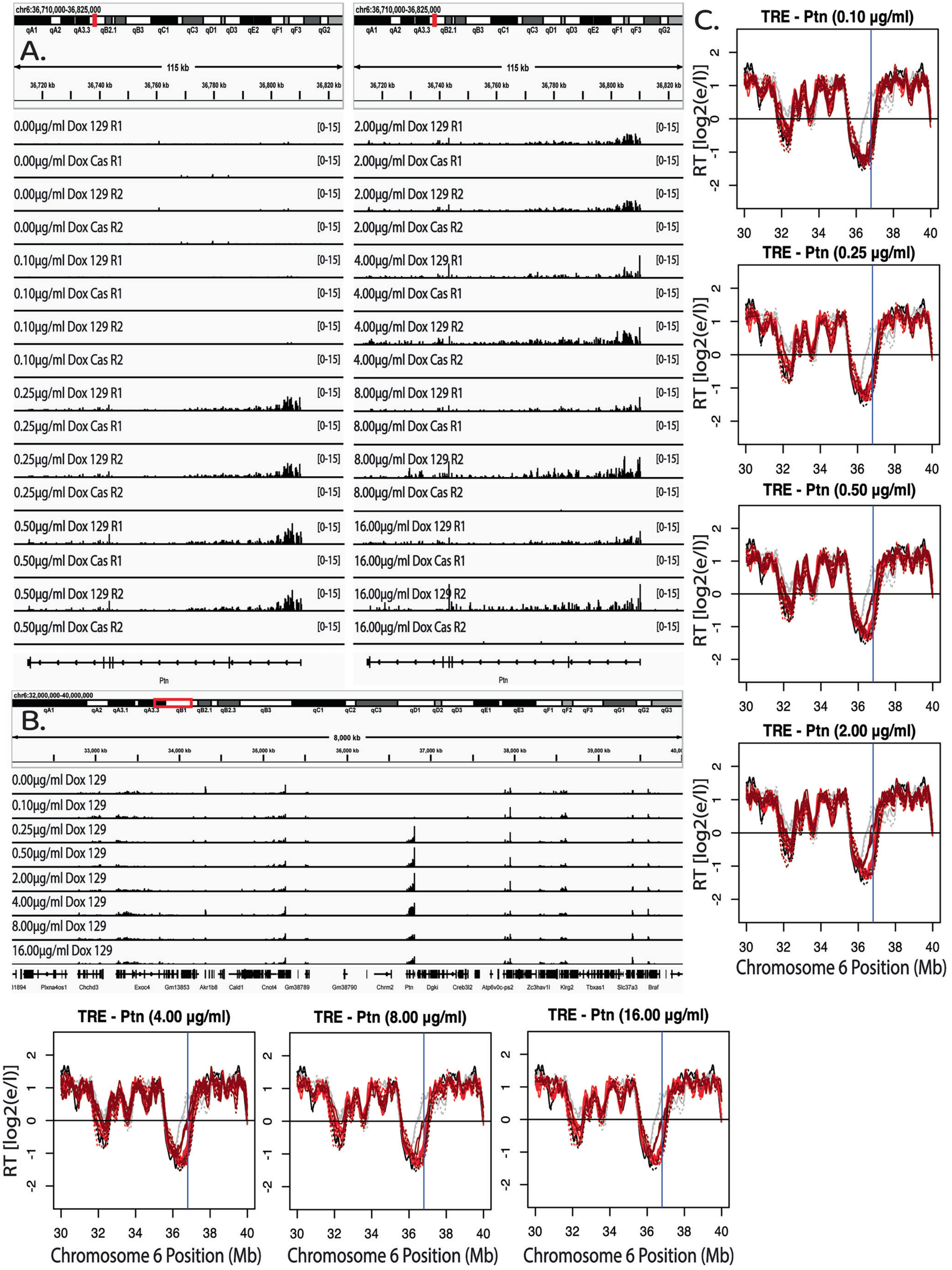
A.
chr6:36,710,000-36,825,000
115 kb
0.00µg/ml Dox 129 R1
0.00µg/ml Dox Cas R1
0.00µg/ml Dox 129 R2
0.00µg/ml Dox Cas R2
0.10µg/ml Dox 129 R1
0.10µg/ml Dox Cas R1
0.10µg/ml Dox 129 R2
0.10µg/ml Dox Cas R2
0.25µg/ml Dox 129 R1
0.25µg/ml Dox Cas R1
0.25µg/ml Dox 129 R2
0.25µg/ml Dox Cas R2
0.50µg/ml Dox 129 R1
0.50µg/ml Dox Cas R1
0.50µg/ml Dox 129 R2
0.50µg/ml Dox Cas R2
2.00µg/ml Dox 129 R1
2.00µg/ml Dox Cas R1
2.00µg/ml Dox 129 R2
2.00µg/ml Dox Cas R2
4.00µg/ml Dox 129 R1
4.00µg/ml Dox Cas R1
4.00µg/ml Dox 129 R2
4.00µg/ml Dox Cas R2
8.00µg/ml Dox 129 R1
8.00µg/ml Dox Cas R1
8.00µg/ml Dox 129 R2
8.00µg/ml Dox Cas R2
16.00µg/ml Dox 129 R1
16.00µg/ml Dox Cas R1
16.00µg/ml Dox 129 R2
16.00µg/ml Dox Cas R2
[0-15]
Ptn
B.
chr6:32,000,000-40,000,000
8,000 kb
0.00µg/ml Dox 129
0.10µg/ml Dox 129
0.25µg/ml Dox 129
0.50µg/ml Dox 129
2.00µg/ml Dox 129
4.00µg/ml Dox 129
8.00µg/ml Dox 129
16.00µg/ml Dox 129
Plxna4os1 Chchd3 Exoc4 Gm13853 Akr1b8 Cald1 Cnot4 Gm38789 Gm38790 Chrm2 Ptn Dgki Creb3l2 Atp6v0c-ps2 Zc3hav1l Klrg2 Tbxas1 Slc37a3 Braf
C.
TRE - Ptn (0.10 µg/ml)
TRE - Ptn (0.25 µg/ml)
TRE - Ptn (0.50 µg/ml)
TRE - Ptn (2.00 µg/ml)
TRE - Ptn (4.00 µg/ml)
TRE - Ptn (8.00 µg/ml)
TRE - Ptn (16.00 µg/ml)
RT [log2(e/l)]
Chromosome 6 Position (Mb)

Figure EV4. Dosage-dependent advance of RT in response to transcriptional induction of the Ptn gene.

(**A**) Genome Browser track of parsed Bru-Seq biological replicates displaying Ptn expression at different concentrations of Dox in the TRE-Ptn cell lines. (**B**) Ten Mb Zoom out of the same Genome Browser tracks in (**A**), demonstrating that the Ptn gene is the only gene whose transcription is affected by Dox induction for 5 Mb upstream or downstream. (**C**) Ten Mb Zoom out of RT across the same regions as in (**B**), demonstrating that the Ptn domain is the only domain whose RT is affected by Dox induction for 5 Mb upstream or downstream.

 © The Author(s)

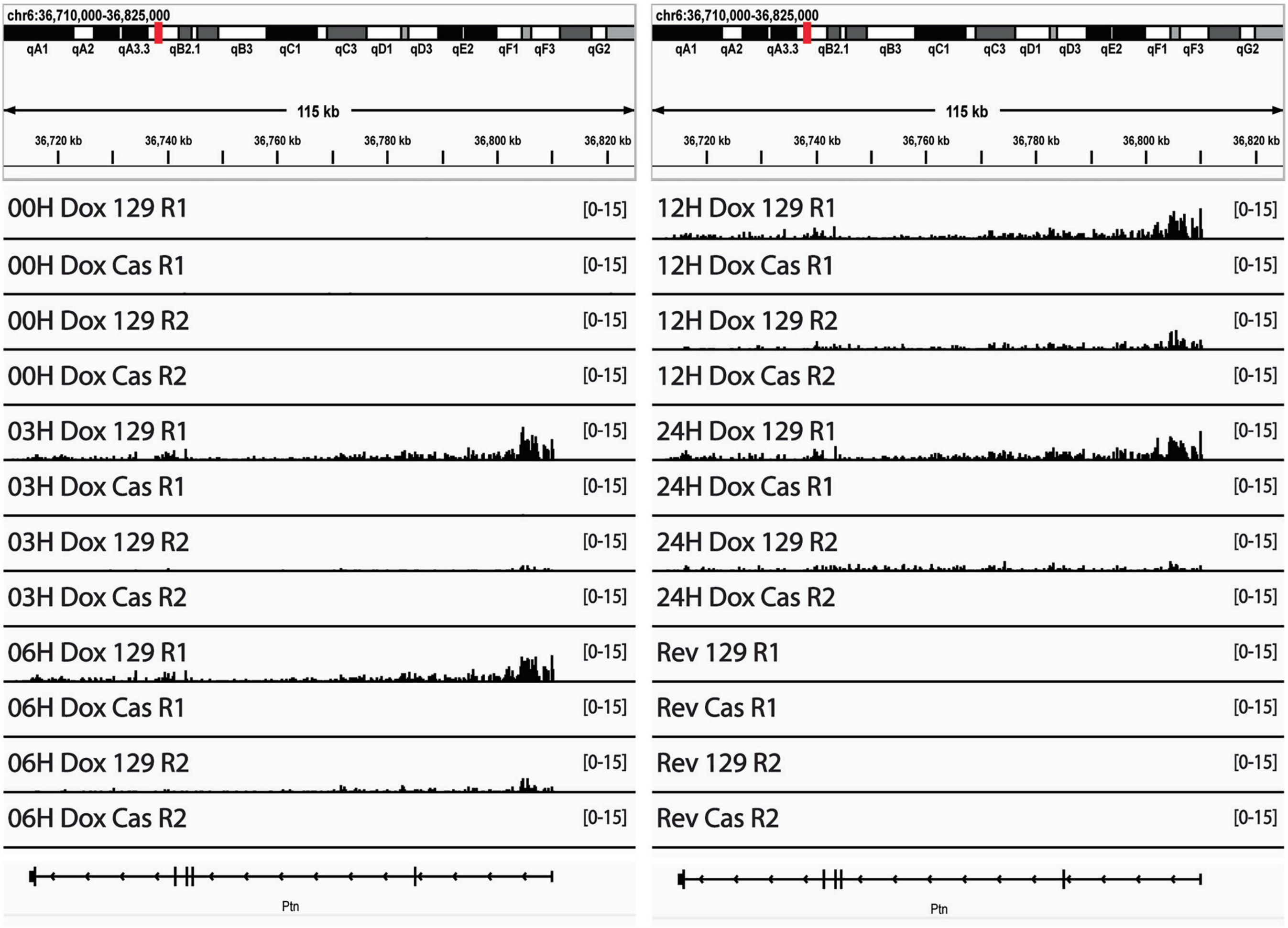

Figure EV5. Genome Browser track of parsed Bru-Seq biological replicates displaying Ptn expression at different time points after the addition of Dox in the TRE-Ptn cell lines.

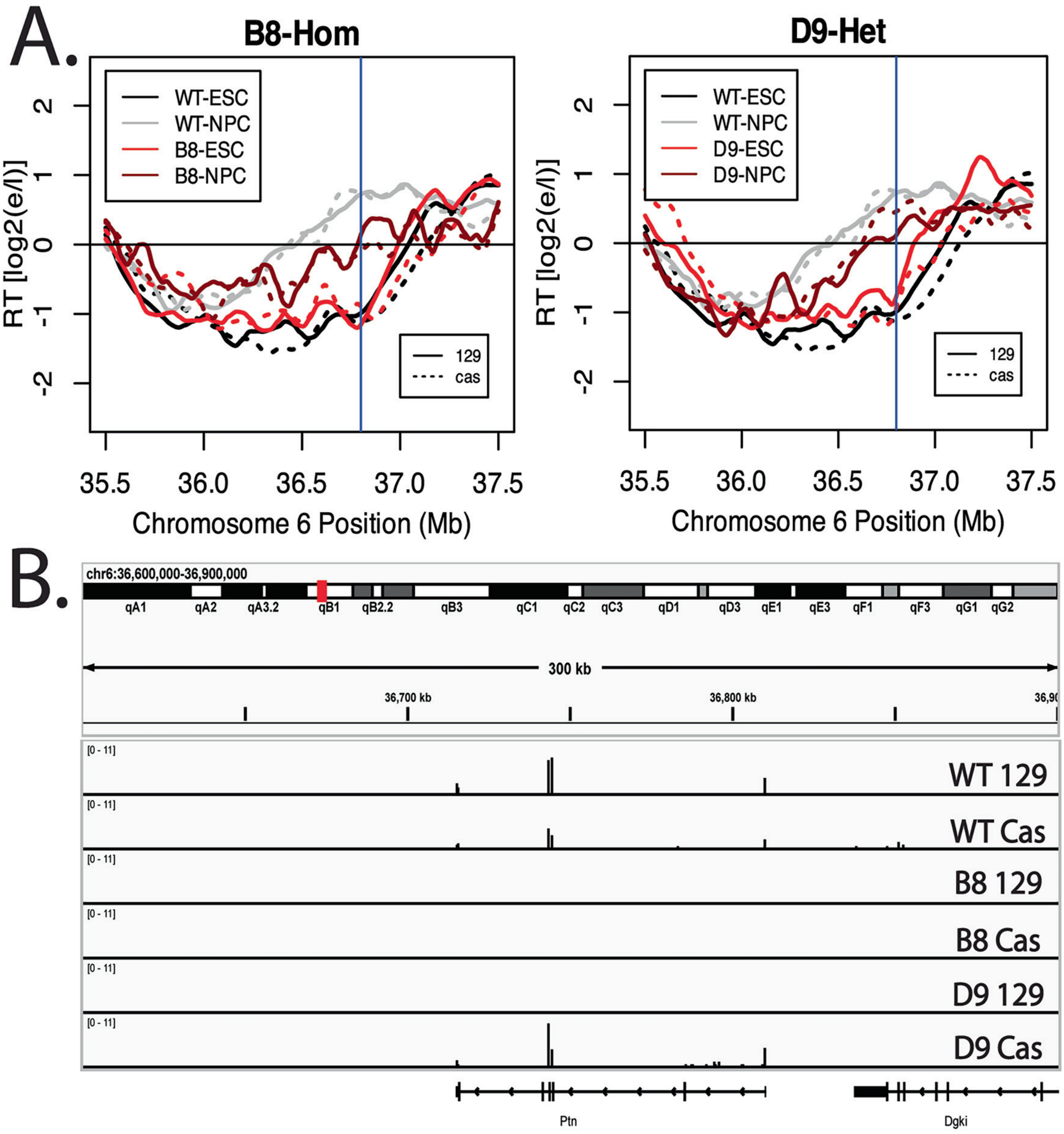

Figure EV6. Deletion of the Ptn promoter and elimination of detectable Ptn trancripts does not significantly affect the RT advance of the Ptn domain during NPC differentiation.

(**A**) RT plots of clones B8 and D9, in which the Ptn promoter deletion is homozygous and Heterozygous, respectively. Dark gray and light gray indicate the RT profiles of WT ESCs and NPCs, respectively. Light red and dark red indicate the RT profiles of ESC and NPC deletion clones, respectively. Solid and dashed lines indicate the RT of the musculus and castaneous alleles, respectively. Vertical line indicates the site of the deletion. (**B**) Browser track of total rRNA-depleted RNA-seq for WT NPCs and the two deletion clones. RNA-seq was performed in lieu of Bru-Seq because cell death during the differentiation process makes it difficult to collect enough cells to perform Bru-Seq.

 © The Author(s)

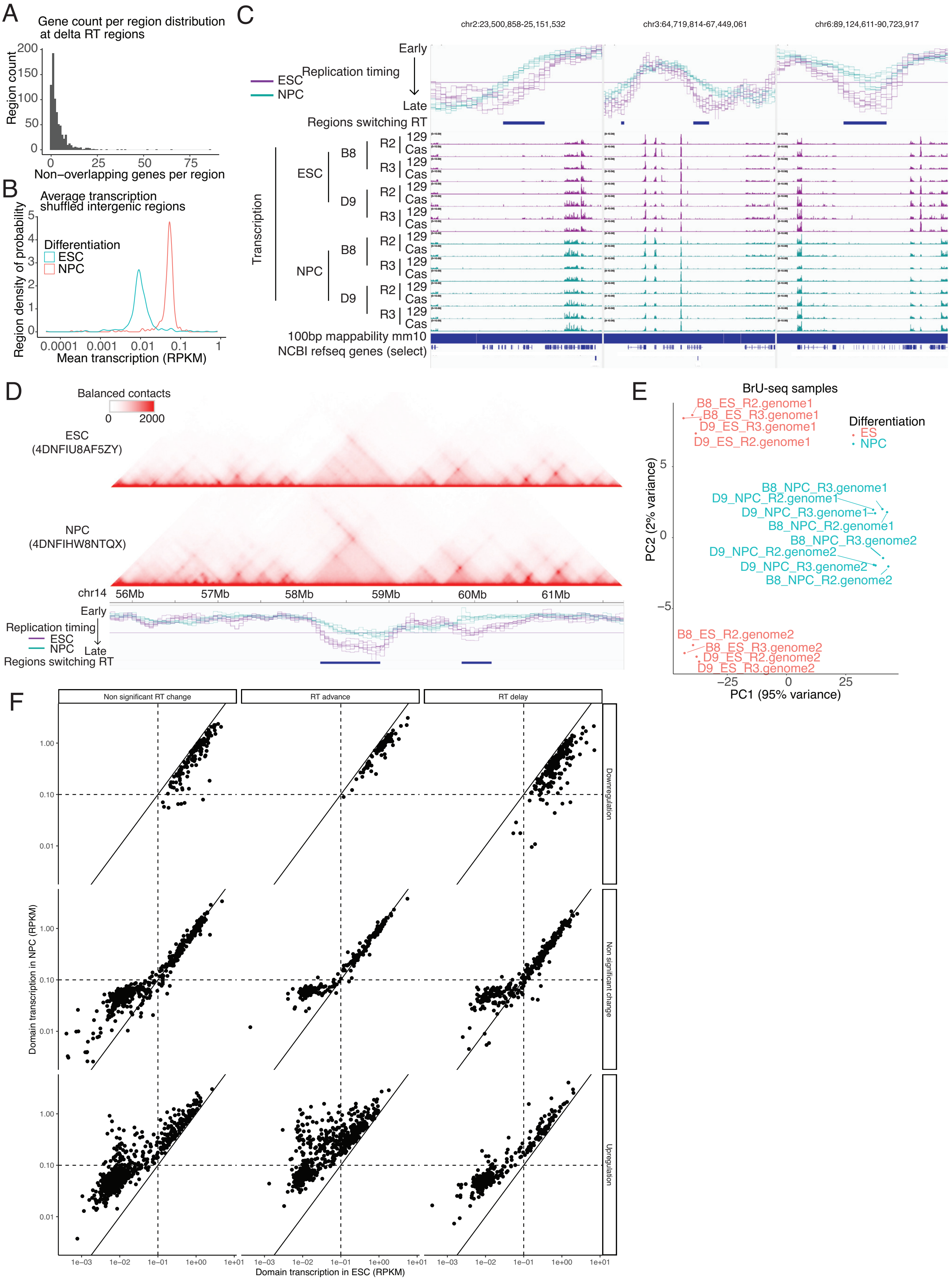
A
Gene count per region distribution at delta RT regions
Region count
200
150
100
50
0
0 25 50 75
Non–overlapping genes per region
B
Average transcription shuffled intergenic regions
Region density of probability
5
4
3
2
1
0
Differentiation
ESC
NPC
0.0001 0.001 0.01 0.1 1
Mean transcription (RPKM)
C
chr2:23,500,858-25,151,532
chr3:64,719,814-67,449,061
chr6:89,124,611-90,723,917
Replication timing
ESC
NPC
Early
Late
Regions switching RT
Transcription
ESC
NPC
B8
D9
R2
R3
129
Cas
100bp mappability mm10
NCBI refseq genes (select)
D
Balanced contacts
0 2000
ESC (4DNFIU8AF5ZY)
NPC (4DNFIHW8NTQX)
chr14 56Mb 57Mb 58Mb 59Mb 60Mb 61Mb
Early
Replication timing
ESC
NPC
Late
Regions switching RT
E
BrU-seq samples
Differentiation
ES
NPC
B8_ES_R2.genome1
B8_ES_R3.genome1
D9_ES_R3.genome1
D9_ES_R2.genome1
B8_NPC_R3.genome1
D9_NPC_R2.genome1
D9_NPC_R3.genome1
B8_NPC_R2.genome1
B8_NPC_R3.genome2
D9_NPC_R2.genome2
D9_NPC_R3.genome2
B8_NPC_R2.genome2
B8_ES_R2.genome2
B8_ES_R3.genome2
D9_ES_R2.genome2
D9_ES_R3.genome2
PC2 (2% variance)
5
0
−5
−25 0 25
PC1 (95% variance)
F
Non significant RT change
RT advance
RT delay
Downregulation
Non significant change
Upregulation
Domain transcription in NPC (RPKM)
1.00
0.10
0.01
1e−03 1e−02 1e−01 1e+00 1e+01
Domain transcription in ESC (RPKM)

© The Author(s)

Figure EV7. Transcription is not necessary and not always sufficient for an RT advance during the mESC to mNPC transition.

(**A**) Histogram presenting the gene count distribution at regions with significant RT changes. More than half of switching regions harbor multiple annotated genes whose transcriptional elongation was summed for subsequent analyses. (**B**) Determination of the transcriptional noise threshold (shown in Fig. 7B,D) by shuffling regions with significant RT advance on the genome, constrained to the intergenic regions only (see Methods). Threshold is set at 0.1 RPKM. (**C**) RT and transcription tracks for three regions with a significant advance in RT with no significant transcription detected during the mESC to mNPC transition. Tracks represent, from top to bottom: Genomic coordinates, RT (log2 ratio Early/Late), regions with significant RT change, transcription coverage (rpm, with a maximum scale set to 10 rpm) in mESC and mNPC datasets, mm10 mappability for 100 bp reads, NCBI RefSeq genes. Data from mESC in purple and data from mNPC are in teal. (**D**) 3D chromatin contacts in ESC (first track) and NPC (second track) at the locus presented in Fig. 7C. RT profile and regions with significant RT changes are plotted below, identical to Fig. 7C, for comparison. (**E**) PCA plot of the BrU-seq gene count demonstrates clear separation of differentiated and undifferentiated cells. (**F**) Dotplot of replication domains. Each dot represents one domain. X-axis represents the average transcription coverage in the region in ESC (read per kb per million reads, RPKM). Y-axis represents the average transcription coverage in the region in NPC (read per kb per million reads, RPKM).

 © The Author(s)

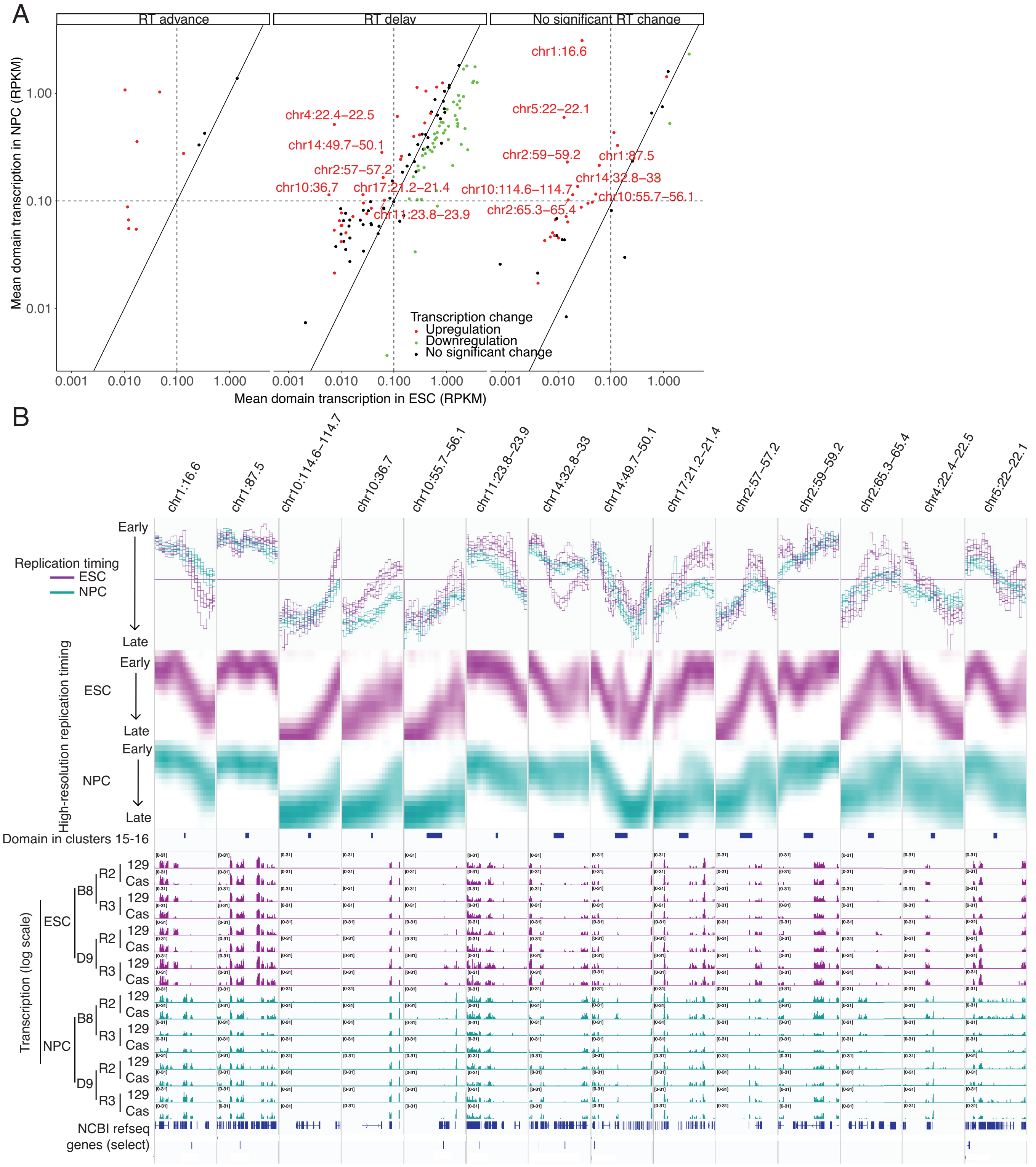
A
RT advance
RT delay
No significant RT change
Mean domain transcription in NPC (RPKM)
Mean domain transcription in ESC (RPKM)
chr1:16.6
chr5:22–22.1
chr4:22.4–22.5
chr14:49.7–50.1
chr2:59–59.2
chr1:87.5
chr2:57–57.2
chr14:32.8–38
chr10:36.7
chr17:21.2–21.4
chr10:114.6–114.7
chr10:55.7–56.1
chr11:23.8–23.9
chr2:65.3–65.4
Transcription change
Upregulation
Downregulation
No significant change
B
Replication timing
ESC
NPC
Early
Late
High-resolution replication timing
Domain in clusters 15-16
Transcription (log scale)
B8
D9
R2
R3
129
Cas
NCBI refseq
genes (select)
chr1:16.6
chr1:87.5
chr10:114.6–114.7
chr10:36.7
chr10:55.7–56.1
chr11:23.8–23.9
chr14:32.8–33
chr14:49.7–50.1
chr17:21.2–21.4
chr2:57–57.2
chr2:59–59.2
chr2:65.3–65.4
chr4:22.4–22.5
chr5:22–22.1

◀ **Figure EV8. Revisiting a prior study identifying genomic regions that induce transcription with no RT advance.**

(**A**) Dotplot of previously identified 200 kb genomic regions containing induced genes that did not advance RT (Hiratani et al, 2010), here re-investigated using Repli-seq and BrU-seq data generated in this manuscript. The X-axis represents the average transcription coverage in the domain in mESCs (read per kb per million reads, RPKM), The Y-axis represents the average transcription coverage in the domain in mNPCs (read per kb per million reads, RPKM). Dot color represents transcription changes of the whole 200 kb window with significant up-regulation (red), significant down-regulation (green), or no significant change (black). Data are separated into three plots by replication timing change within the whole domain (advance, delay or not significant change). (**B**) RT and transcription tracks for the loci around the domains highlighted in (**A**). Tracks represent, from top to bottom: RT (log2 ratio Early/Late), high resolution Repli-seq from WT mESC and for mNPC (GSE137764), original domain localization, transcription coverage (log scale) in mESC and mNPC datasets, NCBI RefSeq genes. Data from mESC are colored in purple and data from mNPC are colored in teal.

 © The Author(s)

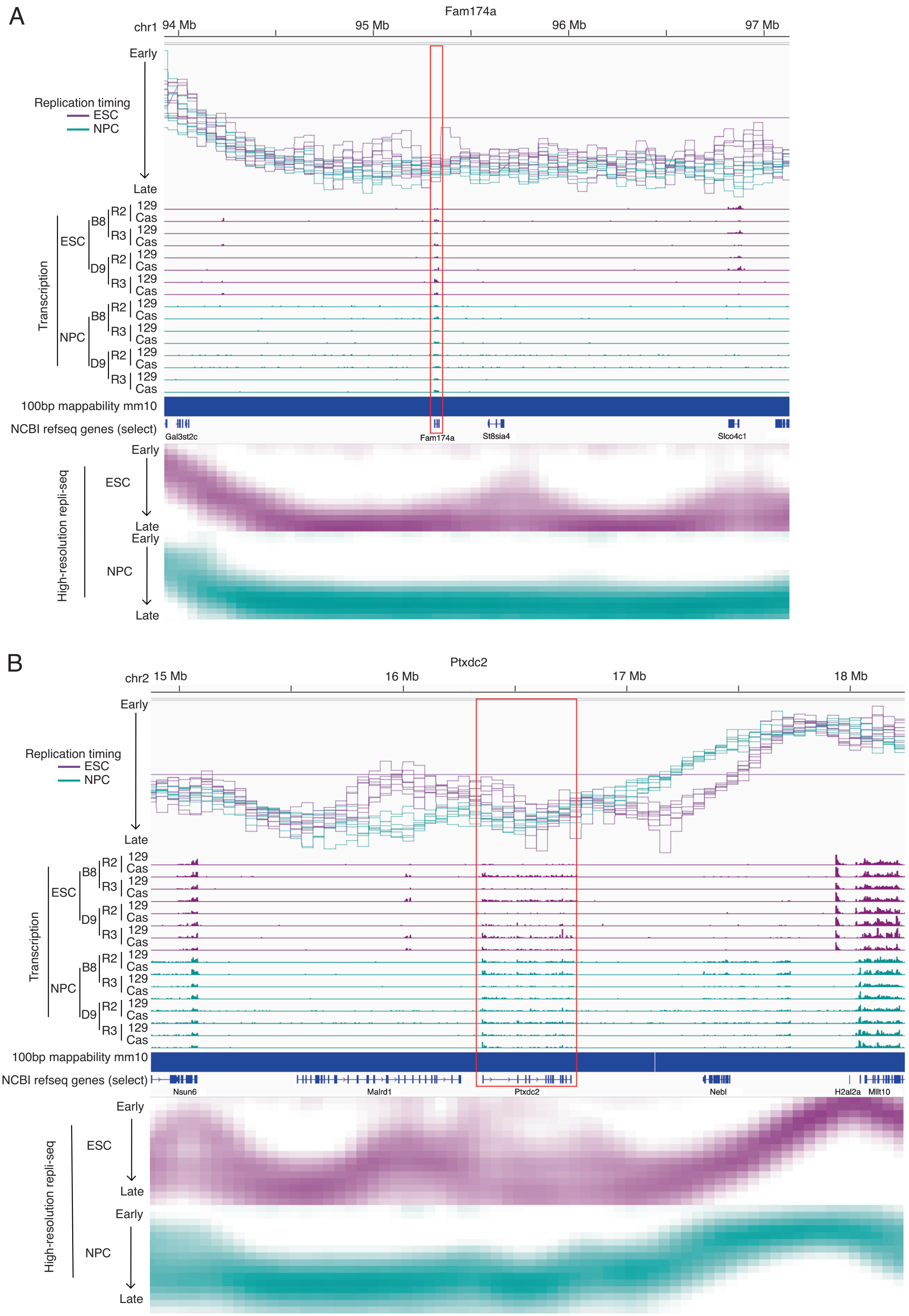
A
Fam174a
chr1 94 Mb 95 Mb 96 Mb 97 Mb
Early
Replication timing
ESC
NPC
Late
Transcription
ESC
NPC
B8
D9
R2
R3
129
Cas
100bp mappability mm10
NCBI refseq genes (select)
Gal3st2c
Fam174a
St8sia4
Slco4c1
High-resolution repli-seq
ESC
NPC
Early
Late
B
Ptxdc2
chr2 15 Mb 16 Mb 17 Mb 18 Mb
Nsun6
Malrd1
Ptxdc2
Nebl
H2al2a
Mllt10

Figure EV9. Early replication is not necessary for transcription.

(**A**, **B**) RT and transcription tracks for the loci around the late-replicating and transcribed genes Fam174a (**A**) and Ptxdc2 (**B**). Tracks represent, from top to bottom: Genomic coordinates, RT (log2 ratio Early/Late), transcription coverage (rpm, with a maximum scale set to 10 rpm) in ESC and NPC, mm10 mappability for 100 bp reads, NCBI RefSeq genes. Data from ESC are colored in purple and data from NPC are colored in teal, high resolution Repli-seq from WT mESC and for mNPC (GSE137764).

 © The Author(s)
